# Effectiveness and Safety of Immunosuppressive Drug Therapy for Neuromyelitis Optica Spectrum Disorders: An Overview of Meta-Analyses and Systematic Reviews

**DOI:** 10.2174/1570159X20666220922151442

**Published:** 2023-06-15

**Authors:** Yuan Luo, Yuqian Deng, Haiye Ran, Lei Yu, Caili Ma, Liping Zhao, Yunchen Li

**Affiliations:** 1 The Second Xiangya Hospital of Central South University, Changsha, Hunan Province, 410011, China;; 2 Xiangya School of Nursing, Central South University, Changsha, Hunan Province, 410013, China;; 3 Xiangya School of Medicine, Central South University, Changsha, Hunan Province, 410013, China

**Keywords:** Immunosuppressive drug therapy, neuromyelitis optica spectrum disorders NMOSD, drug effectiveness and safety, overview, GRADE, central nervous system

## Abstract

***Objective*:** This study aims to provide an overview of meta-analyses and systematic reviews on the effectiveness and safety of immunosuppressive drug therapy for neuromyelitis optica spectrum disorders (NMOSD) by evaluating the methodological quality and reporting quality of reviews.

***Methods*:** The Chinese National Knowledge Infrastructure (CNKI), WanFang Data, China Science and Technology Journal Database, Web of Science, the Cochrane Library, PubMed, and Embase databases were searched to collect systematic reviews or meta-analyses on the effectiveness and safety of immunosuppressive therapy for NMOSD from inception to December 2, 2021. Two researchers independently screened reviews and extracted data. Any differences in the procession of review assessment between the two researchers were re-evaluated, and the disagreement was resolved by discussion with other researchers. The following data were extracted: author, year of publication, the country where the study was conducted, study type, the number of included studies, sample size, risk bias tools, medication of immunosuppressive therapy, and main outcomes. Then, the AMSTAR-2, which is a critical appraisal tool for systematic reviews (2^nd^ edition), and Grades of Recommendation, Assessment, Development and Evaluation (GRADE) were used to evaluate the methodological quality and reporting quality of evidence. A comprehensive analysis was conducted on the outcomes for all included reviews.

***Results*:** A total of 15 reviews were included. Of the included reviews, 3 were systematic reviews, 7 were meta-analyses, and 5 were systematic reviews and meta-analyses. According to the AMSTAR-2 criteria, 6 studies had high quality, 1 study had moderate quality, 4 studies had low quality, and 4 studies had critically low quality. Based on the GRADE, neither evidence quality for effectiveness nor safety was high.

***Conclusion*:** Immunosuppressive drug therapy is effective for patients with NMOSD, but its safety is controversial. Due to the poor quality of evidence, reliability needs to be considered. Thus, large sample, multi-center, double-blind, randomized controlled studies are still needed in the future.

## 1 INTRODUCTION

Neuromyelitis optica spectrum disorder (NMOSD), previously known as Devic's disease, is an inflammatory demyelinating disease of the central nervous system. It is an autoimmune disorder that primarily affects the optic nerve [1]. It is related to humoral immunity and antibodies against Aquaporin-4 (AQP4). These antibodies can bind to AQP4 channels on astrocytes and activate the classical complement cascade, resulting in infiltration of granulocytes, eosinophils, and lymphocytes, followed by oligodendrocyte damage and neuronal death [2, 3]. The main symptoms are acute optic neuritis and transverse myelitis. NMOSD occurs more commonly in non-white populations. Especially, Asian or African populations, females, and people over 35 years of age are at high risk for NMOSD [4, 5]. Relapse, disability, and even death are common outcomes of NMOSD. Patients with NMOSD frequently suffer severe relapses resulting in permanent disability, and thus suppressing relapse frequency and severity is a primary goal in disease management [6].

At present, immunosuppressive drugs are clinically recommended for the treatment of NMOSD due to their effectiveness in preventing relapse, especially during maintenance treatment. The first-line drugs, such as azathioprine (AZA), mycophenolate mofetil (MMF), and rituximab (RTX), are widely used. Recently, other drugs, such as mitoxantrone (MTX) and monoclonal antibody (mAb) (including Eculizumab, Inebilizumab, Satralizumab, and Tocilizumab (TCZ)) have been developed [7-9]. However, there are many controversies on immunosuppressive therapy in clinical treatment. For example, AZA, a traditional drug, has longer onset and more adverse effects. Therefore, in recent years, the effectiveness and safety of more drugs have been explored [10].

Meta-analyses and systematic reviews are essential to summarize evidence on the efficacy and safety of healthcare interventions. They rank as the highest level of evidence in the evidence grading system issued by major centers of evidence-based medicine. They also serve as the basis for developing recommendations and clinical practice guidelines. These reports, however, are less transparent and clear on the effectiveness and safety of immunosuppressive drug therapy for NMOSD. Poor reporting quality diminishes their value to clinicians, policymakers, and other users [11, 12]. Overview of reviews is a method of comprehensively collecting meta-analyses and systematic reviews of the etiology, diagnosis, treatment, and prognosis of the same disease or health problem for re-assessment. It provides a comprehensive amount of high-quality evidence for the users to apply knowledge and evidence [13].

The paper aims to investigate the effectiveness and safety of immunosuppressive drug therapy for NMOSD by summarizing and evaluating meta-analyses and systematic reviews. In the future, our findings may provide references for the clinical treatment of NMOSD.

## 2 METHODS

### 2.1 Study Protocol

We designed a study protocol according to the Cochrane Collaboration format at the beginning of the study. This overview was compiled to the Preferred Reporting Items for Systematic Reviews and Meta-Analyses (PRISMA) and was registered in the international prospective register of systematic reviews (PROSPERO) (CRD42022295949).

### 2.2 Study Selection

The Chinese National Knowledge Infrastructure (CNKI), WanFang Data, China Science and Technology Journal Database, Web of science, the Cochrane Library, PubMed, and Embase databases were searched to collect systematic reviews or meta-analyses on the effectiveness and safety of immunosuppressive therapy for NMOSD from inception to December 2, 2021. The following words were used in the search strategies: neuromyelitis optica spectrum disorder, neuromyelitis optica, Devic Disease, NMOSD, NMO, Immunosuppressive agents, Azathioprine (AZA), Mycophenolate Mofetil (MMF), Rituximab (RTX), Imuran, Tocilizumab, Monoclonal Antibodies (mAb), Anti-IL-6, interleukin-6-receptor inhibitors, Meta-analysis, and, Systematic Review. Fig. (**1**) shows the search strategies in PubMed. The search terms should be expanded and supplemented based on the search results.

### 2.3 Inclusion and Exclusion Criteria for Reviews

The full texts of the retrieved articles were assessed for eligibility. According to the PICOS principle, the inclusion criteria were as follows: (1) Population (P): Patients with NMOSD. (2) Intervention (I): Treatment with immunosuppressive drugs. (3) Control (C): different immunosuppressive and non-immunosuppressive treatments. (4) Outcomes (O): annualized relapse rate (ARR); relapse risk or first relapse; extended disability status scale (EDSS); relapse-free rate (RFR); adverse events or serious adverse events; death or mortality. (5) Study type (S): meta-analyses or systematic reviews; (6) Languages were restricted to Chinese or English. We excluded reviews based on the following exclusion criteria: (1) Duplicate publications; (2) Protocols for systematic review and meta-analysis or in the research and design stage; (3) Network meta-analyses; (4) Conference summaries or incomplete reviews; (5) Full text was not found.

### 2.4 Data Extraction

Two researchers independently extracted the data, and any disagreements were resolved by group discussion. The following information was extracted from the reviews: the first author, published year, country, study type, number of included studies, sample size, risk bias tools, medication of immunosuppressive therapy, and main outcomes.

### 2.5 Quality Assessment

AMSTAR-2, which is a critical appraisal tool for systematic reviews (2nd edition), was revised based on the first version in 2017 by researchers from Dutch and Canadian research institutions who specialize in clinical epidemiology and evidence-based medicine. It is currently recognized worldwide and used widely [14]. The methodology quality of the included reviews was evaluated by two researchers independently with the AMSTAR-2 tool. In total, 16 items were assessed, and seven of them were critical (including item 2, item 4, item 7, item 9, item 11, item 13, and item 15). The evaluation of each item has three options, *i.e*., “Yes”, “Partial Yes” and “No”. If less than or equal to one non-critical item is not met, high quality was defined; if more than one non-critical item is not met, medium quality was defined; if a critical item with or without non-critical items is not met, low quality was defined; and, if more than one critical item is not met, lower quality was defined. According to the criteria, AMSTAR-2 classifies the overall confidence levels in the assessment results into four levels: high, moderate, low, and critically low [15]. Disagreements between the two researchers were resolved through discussion with other researchers.

We used the Grades of Recommendation, Assessment, Development and Evaluation (GRADE) to assess the strength of evidence associated with specific outcomes. Five factors (*i.e*., bias risk, inconsistency, indirectness, imprecision, and publication bias) may lead to rating down the quality of evidence and three factors (*i.e*., large effect, dose response and all plausible confounding) may lead to rating up the quality of evidence. The levels of evidence are divided into high, moderate, low, and very low [16].

### 2.6 Evidence Analysis

We carried out descriptive analyses of the quality of the included reviews. We further described and assessed the summary evidence of immunosuppressive therapy for NMOSD. Finally, we summarized the study findings.

## 3 RESULTS

### 3.1 Study Identification and Selection

**Fig. (2):**
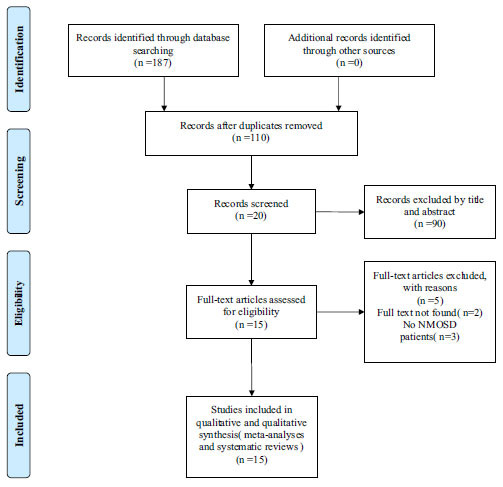
PRISMA flow chart of study selection according to Preferred Reporting Items for Systematic Reviews and Meta-Analyses.

In the initial search, 187 reviews were retrieved from the database. After removing duplicate publications, there were 110 reviews. Following screening the title and abstract, 20 reviews were obtained. Finally, 15 reviews were eligible after assessing full texts (Fig. **2**).

### 3.2 Descriptions and Characteristics of Included Reviews

Of the included reviews, 3 were systematic reviews, 7 were meta-analyses, and 5 were systematic reviews and meta-analyses. The year of publication was from 2016 to 2021. The medication of immunosuppressive therapy included AZA, MTX, MMF, and mAb (including TCZ, Eculizumab, Inebilizumab, RTX, and Satralizumab). We paid more attention to the effectiveness and safety of immunosuppressive drug therapy. Based on the analysis of the included reviews, ARR, EDSS, and RFR were the main outcomes of effectiveness. Adverse events and occurrence rates were the main outcomes of safety (Table **1**).

### 3.3 Quality of Included Reviews

The overall quality of the included reviews is summarized in Table **2**. Six studies were of high quality; 1 study was of moderate quality; 4 studies were of low quality; 4 studies were of critically low quality. This result indicates that the included meta-analyses or systematic reviews have less than good quality.

#### 3.3.1 The Items of AMSTAR-2

1. Did the research questions and inclusion criteria for the review include the components of PICO? 2. Did the report of the review contain an explicit statement that the review methods were established prior to the conduct, and did the report justify any significant deviations from the protocol? 3. Did the review authors explain their selection of the study designs for inclusion in the review? 4. Did the review authors use a comprehensive review search strategy? 5. Did the review authors perform study selection in duplicate? 6. Did the review authors perform data extraction in duplicate? 7. Did the review authors provide a list of excluded studies and justify the exclusions? 8. Did the review authors describe the included studies in adequate detail? 9. Did the review authors use a satisfactory technique for assessing the risk of bias (RoB) in individual studies included in the review? 10. Did the review authors report on the sources of funding for the studies included in the review? 11. If meta-analysis was performed, did the review authors use appropriate methods for statistical combination of results? 12. If meta-analysis was performed, did the review authors assess the potential impact of RoB in individual studies on the results of the meta-analysis or other evidence synthesis? 13. Did the review authors account for RoB in primary studies when interpreting/discussing the results of the review? 14. Did the review authors provide a satisfactory explanation for, and discussion of, any heterogeneity observed in the results of the review? 15. If they performed quantitative synthesis, did the review authors carry out an adequate investigation of publication bias (small study bias) and discuss its likely impact on the results of the review? 16. Did the review authors report any potential sources of conflict of interest, including any funding they received for conducting the review?

### 3.4 Quality Assessment of the Evidence

The results of the GRADE showed that none of the evidence quality was high. Most of the evidence levels were low or very low. There were two main factors that affected the quality: the lack of randomized controlled trials (RCTs) in the included reviews and the absence of assessment for publication bias. The assessment outcomes of each piece of evidence are shown in Table **3**. All in all, the level of evidence on effectiveness and safety was not ideal.

## LIMITATION

Meta-analyses and systematic reviews included in this study were heterogeneous and quantitative analysis could not be performed, which may result in some bias. Most of the included reviews acknowledged the poor quality of the included original studies, which may influence the reliability of the results.

## CONCLUSION

### Effectiveness of Immunosuppressive Drug Therapy

The primary therapy goal for patients with NMOSD is to prevent disease relapse and disability. In general, immunosuppressive drug therapy is effective for patients with NMOSD. Comparisons of immunosuppressive drugs, especially first-line drugs, have attracted much attention. Espiritu *et al.* suggested that AZA may be inferior to RTX in terms of reduction of ARR, RFR, and EDSS [18]. Velasco *et al.* also suggested that RTX was confirmed to have better efficacy compared to other drugs [25]. Giovannelli *et al.* suggested that AZA, RTX, and MMF had similar effects on disease relapse, but RTX might be more efficacious than AZA in controlling the clinical symptoms [20]. RTX, as one of the mAb drugs, is widely applied to NMOSD patients. The effectiveness of RTX treatment may be associated with the duration of the disease, body mass index, and the type of immunosuppressive drug used before RTX treatment [26]. As research progresses, the new drugs eculizumab, inebilizumab, satralizumab, and TCZ have shown high efficacy for maintenance treatment [21, 24, 25, 28]. Especially, AQP4-positive NMOSD patients show higher efficacy in relapse and disability prevention, but this benefit is not found in AQP4-negative NMOSD patients [21, 22, 29]. Among mAb drugs, eculizumab is likely to be more effective at preventing recurrence in AQP4-positive patients [29]. Early drug therapy is critical for disability prevention. The earlier treatment may reduce disability more effectively [17, 26].

### Safety of Immunosuppressive Drug Therapy

Safety is also an important aspect of maintenance therapy by immunosuppressive drugs. However, the results on drug safety are controversial. The adverse events and incidence rate have been widely studied. A study showed that there was a significant number of patients in the AZA group who had experienced adverse events, such as infection, leukopenia, and hair loss, compared to that in the MMF and RTX groups [19]. In contrast, other studies showed that AZA could frequently cause adverse events [23, 25], but drug-related complications were generally minor and complications of NMOSD were responsible for most deaths [5, 17, 23]. We need to observe and distinguish carefully to make targeted treatments. Recently, mAb therapy has been confirmed to reduce adverse events. NMOSD can be treated with RTX, a chimeric mAb directed against the surface antigen of CD20 on B lymphocytes, which is highly recommended and widely applied [31, 32]. Other mAb drugs, including eculizumab, inebilizumab, satralizumab, and TCZ, have also been widely studied. However, their safety is in dispute. No significant differences were observed between the mAb group and placebo group in adverse events and mortality. More RCTs with large sample sizes are needed for each mAb [21, 22, 28, 29].

### Poor Quality of Evidence

Meta-analyses and systematic reviews are considered to be the best evidence integration method, but the conclusions should be treated with caution due to the influence of methodological quality and the quality of included original studies [12]. As a result, physicians may be hesitant to use immunosuppressive drugs in the treatment. First, none of the reviews mentioned the sources of funding for the studies included in the review. Studies funded by industry were less likely to be published than those funded by non-industry sectors [15]. Secondly, half of the included reviews did not assess publication bias. Authors may find it difficult to eliminate publication bias, but it is an important issue [12, 15]. Finally, there was no high-quality evidence of effectiveness or safety. The main reason is the lack of RCTs. The included 15 meta-analyses and systematic reviews mainly consisted of observational studies and case reports, which may lead to low quality of original evidence. Although some studies include adequate samples with different doses, it is so difficult to upgrade level of the evidence, due to other uncontrolled confounding factors. Thus, large sample, multi-center, double-blind RCTs are still needed in the future.

## Figures and Tables

**Fig. (1):**
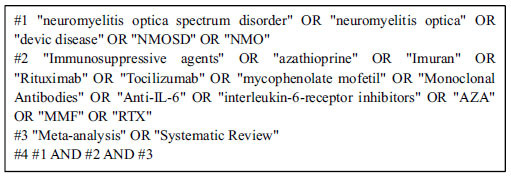
The search strategies in PubMed.

**Table 1:** Descriptions and characteristics of included reviews.

**First Author, ** **Publication Year/Refs.**	**Country**	**Study Type**	**Study ** **Number**	**Sample ** **Size**	**Risk Bias ** **Tools**	**Medication of Immunosuppressive Therapy**	**Outcomes**
Damato 2016 [17]	Italy	Systematic Review and Meta-analysis	46	438	/	RTX	①②④
Enriquez 2019 [18]	Philippines	Systematic Review	8	117	(1)	MTX	①②③④⑤
Espiritu 2019 [19]	Philippines	Systematic Review and Meta-analysis	9	977	(1)(2)	AZA	①②③④⑤
Giovannelli 2021 [20]	France	Meta-analysis	7	919	(1)(5)	AZA, MMF, RTX	①
Kharel 2021 [5]	Nepal	Meta-analysis	9	202	(1)(2)(5)	Satralizumab, TCZ	①②④⑤
Kong 2021 [21]	China	Meta-analysis	7	603	(1)(2)(5)	Eculizumab, Inebilizumab, RTX, Satralizumab, TCZ	①②④
Lotan 2021 [22]	USA	Systematic Review	25	424	/	Satralizumab, TCZ	①②④⑤
Luo 2020 [23]	China	Meta-analysis	21	1016	(1)(5)	AZA	①②③④⑤
Songwisit 2020 [24]	Thailand	Systematic Review and Meta-analysis	15	1047	(1)	MMF	①②④
Velasco 2021 [25]	Colombia	Systematic Review	13	1447	(3)(4)	AZA, RTX, MMF, mAb	①②④
Wang 2021 [26]	China	Systematic Review and Meta -analysis	29	732	(5)	RTX	①②
Xie 2020 [27]	China	Meta-analysis	5	89	/	TCZ	①②③④
Xue 2020 [28]	China	Meta-Analysis	7	775	(2)	Eculizumab, Inebilizumab, RTX, Satralizumab, TCZ	①②④⑤
Xue 2020 [29]	China	Meta-analysis	4	524	(2)	Eculizumab, Inebilizumab, RTX, Satralizumab, TCZ	①②④⑤
Wang 2021 [30]	China	Systematic Review and Meta-analysis	14	930	(1)(5)	MMF	①

**Note: Medication of immunosuppressive therapy:** AZA: Azathioprine; MTX: Mitoxantrone; MMF: Mycophenolate Mofetil; mAb: Monoclonal Antibody, including Eculizumab, Inebilizumab, Rituximab (RTX), Satralizumab, Tocilizumab (TCZ).

**Risk bias tools:** (1) Newcastle-Ottawa Quality Assessment Tool; (2) the Cochrane Collaboration Tool; (3) SIGN tool; (4) ROBINS tool; (5) Egger test or funnel plots.

**Outcome:** ① Annualized relapse rate (ARR), Relapse risk or First relapse; ② Extended Disability Status Scale (EDSS); ③ Relapse free rate (RFR); ④ Adverse events or serious adverse events; ⑤ Death or Mortality.

**Table 2:** The methodology quality of the included reviews.

**Author, ** **Publication Year**	**AMSTAR-2 Items**																**Quality Rank**
	1	2	3	4	5	6	7	8	9	10	11	12	13	14	15	16	
Damato 2016	Y	N	N	PY	Y	Y	Y	PY	N	N	Y	Y	Y	Y	N	Y	Critically low
Enriquez 2019	Y	Y	Y	Y	Y	N	PY	Y	Y	N	/	/	N	Y	N	Y	Critically low
Espiritu 2019	Y	Y	Y	PY	N	N	Y	Y	Y	N	Y	Y	Y	Y	N	Y	Low
Giovannelli 2021	Y	Y	Y	Y	Y	Y	Y	Y	Y	N	Y	Y	Y	Y	Y	Y	High
Kharel 2021	Y	Y	N	Y	Y	Y	Y	Y	Y	N	Y	Y	Y	Y	Y	Y	High
Kong 2021	Y	Y	Y	Y	Y	Y	Y	Y	Y	N	Y	Y	Y	Y	Y	Y	High
Lotan 2021	N	PY	N	PY	N	N	PY	Y	N	N	/	/	N	N	/	Y	Critically low
Luo 2020	Y	Y	Y	PY	Y	Y	Y	Y	Y	N	Y	Y	Y	Y	Y	Y	High
Songwisit 2020	Y	Y	Y	PY	Y	Y	Y	Y	Y	N	Y	Y	Y	Y	N	Y	Low
Velasco 2021	Y	Y	Y	PY	Y	Y	Y	Y	Y	N	/	/	Y	Y	/	Y	Moderate
Wang 2021	Y	Y	Y	PY	Y	Y	Y	PY	Y	N	Y	Y	Y	Y	Y	Y	High
Xie 2020	Y	Y	Y	PY	Y	Y	PY	PY	N	N	Y	Y	Y	Y	N	Y	Critically low
Xue 2020	Y	Y	Y	Y	Y	Y	Y	Y	Y	N	Y	Y	Y	Y	N	Y	Low
Xue 2020	Y	Y	Y	Y	Y	Y	Y	PY	Y	N	Y	Y	Y	Y	N	Y	Low
Wang 2021	Y	Y	Y	PY	Y	Y	Y	PY	Y	N	Y	Y	Y	Y	Y	Y	High

**Note**: Y: Yes; PY: Partial Yes; N: NO.

**Table 3:** Quality assessment of effectiveness and safety evidence.

**Author, Publication Year**	**Effectiveness**			**Safety**	
	**ARR**	**EDSS**	**RFR**	**AE**	**Death**
Damato 2016	Low	Low	/	Very low	/
Enriquez 2019	Very low	Very low	Very low	Very low	Very low
Espiritu 2019	Very low	Very low	Very low	Moderate	/
Giovannelli 2021	Moderate	/	/	/	/
Kharel 2021	Low	Low	/	Low	Very low
Kong 2021	Moderate	Moderate	/	Low	/
Lotan 2021	Very low	Very low	/	Very low	Very low
Luo 2020	Moderate	Moderate	Moderate	Low	Very low
Songwisit 2020	Low	Very low	/	Very low	/
Velasco 2021	Low	Low	/	Very low	/
Wang 2021	Low	Low	/	/	/
Xie 2020	Very low	Very low	Very low	Very low	/
Xue 2020	Moderate	Moderate	/	Moderate	Moderate
Xue 2020	Low	Very low	/	Low	Very low
Wang 2021	Moderate	/	/	/	/

**Note**: /: Not mentioned. **Abbreviations**: ARR: annualized relapse rate; EDSS: extended disability status scale; RFR: relapse free rate; AE: Adverse event; Death: death or mortality.

## LIST OF ABBREVIATIONS

AQP4: Aquaporin-4
AZA: Azathioprine
EDSS: Extended Disability Status Scale
MMF: Mycophenolate Mofetil
MTX: Mitoxantrone
NMOSD: Neuromyelitis Optica Spectrum Disorder
PRISMA: Preferred Reporting Items for Systematic Reviews and Meta-Analyses
RCTs: Randomized Controlled Trials
RFR: Relapse-free Rate
RoB: Risk of Bias
RTX: Rituximab

## STANDARDS OF REPORTING

PRISMA guidelines and methodology were followed.
